# Editorial: Affiliative touch and sense of self: theoretical foundations and innovative treatments

**DOI:** 10.3389/fpsyg.2025.1620556

**Published:** 2025-05-16

**Authors:** Maurizio Peciccia, Vittorio Gallese, Mark Solms

**Affiliations:** ^1^Department of Philosophy, Social Sciences, Humanities and Education, University of Perugia, Perugia, Italy; ^2^Department of Medicine and Surgery, University of Parma, Parma, Italy; ^3^Neuroscience Institute and Department of Psychology, University of Cape Town, Cape Town, South Africa

**Keywords:** Touch Medicine, C-T fiber stimulation, HPA axis regulation and dysregulation, attachment regulation and dysregulation, bodily self formation, embodied simulation regulation and dysregulation

Affiliative touch is widely recognized as a foundational component of interpersonal sensorimotor interactions, critically underpinning the formation and maintenance of social bonds (Insel and Young, 2001; Kosfeld et al., 2005) as well as the development of a coherent sense of self (Bowlby, 1988; Ainsworth, 1989; Beebe et al., 2010), processes that are essential for survival and adaptive capacity (Dunbar, 2010). At the core of these processes, the C-tactile (C-T) system (C-T receptors and fibers) mediates affiliative touch by transmitting signals generated from gentle stroking stimuli to a diverse network of brain regions spanning subcortical, cortical, and intercortical/intersubjective levels (Walker et al., 2017).

**At the subcortical level**, the C-T fibers' projections to the hypothalamus and the limbic system modulate the stress response (Neumann, 2002; Kidd et al., 2023) and facilitate attachment (Feldman, 2012; Walker and McGlone, 2013; Feldman et al., 2010). Furthermore, by stimulating oxytocin synthesis, affiliative touch regulates corticosteroid hormones (Heinrichs et al., 2009), thereby influencing glial cell function (Sierra et al., 2008). This modulation may, in turn, affect dopaminergic neurotransmission in stress-sensitive subcortical regions, as we all as the hippocampus and neocortical areas, such as the prefrontal cortex (Sierra et al., 2008; Howes and McCutcheon, 2017).

**At the cortical level**, afferent pathways linking C-T receptors and fibers to the posterior insula (Olausson et al., 2002) contribute to constructing a bodily self-mapping by integrating interoceptive signals with exteroceptive inputs from the posterior parietal cortex (Crucianelli and Filippetti, 2020; McGlone et al., 2014). This integrative multisensory process, together with coordinated activity in motor cortical areas and subcortical networks, is central to delineating self-boundaries and establishing bodily ownership, both of which are fundamental for maintaining a healthy sense of self (Craig, 2009; Gallese et al., 2024; Gallese and Sinigaglia, 2010, 2011; Blanke, 2012; Serino et al., 2013; Tsakiris, 2010).

**At intercortical/intersubjective level**, affective touch, by reinforcing the distinction between self and other, regulates embodied simulation. This process relies on two concurrent principles: one of similarity, whereby the other is implicitly perceived as an extension of the self, and one of differentiation, which preserves the critical boundary between self and other (Gallese, 2003, 2005, 2007, 2014).

This Research Topic of articles on “*Affiliative touch and sense of self: theoretical foundations and innovative treatments*” aspires to encourage clinical trials and large-scale studies on affective touch as a natural, safe, and cost-effective intervention. The objective of this Research Topic is to support inclusion of affiliative touch in national and international guidelines addressing psychological distress related to disturbances of the sense of self.

McGlone et al. introduce the concept of “Touch Medicine” as an interdisciplinary framework aimed at bridging the gap between extensive basic research on affective touch and its limited clinical implementation. The authors highlight the association between a lack of touch in childhood and adverse health outcomes, and they present evidence supporting the effectiveness of therapeutic touch in preventing and treating various conditions. They draw attention to studies and reviews that demonstrate the efficacy of massage in the treatment of depression, as well as controlled studies that support the use of touch interventions for the alleviation of symptoms associated with depression, anxiety and pain. This may occur through interoceptive, endocrine and stress-related pathways. The authors emphasize the urgent need for large-scale therapeutic protocols based on affiliative touch to improve both treatment and prevention strategies.

Papi et al. conducted a systematic review in accordance with PRISMA guidelines of recent research on C-T fiber stimulation through affective touch. The review focused on two areas: assessing C-T fiber dysregulation in psychological disorders and examining C-T fiber based therapies. The results showed that people with psychological disorders often exhibit sensory differences, frequently rating affective touch as less pleasant than healthy people. Although the number of empirical studies on the therapeutic applications of affective touch is comparatively limited in relation to its basic research, there is a consensus among researchers that these therapies show promise in reducing symptom severity and improving interoception in various psychological conditions. However, it is acknowledged that further research is required to substantiate these conclusions.

**Figure 1:**
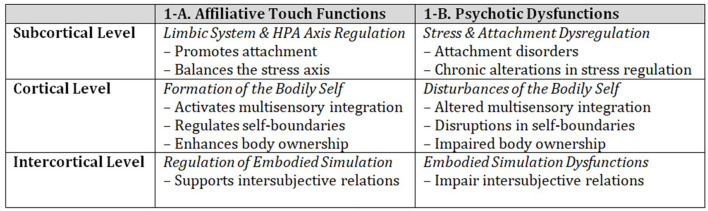
Affiliative touch functions and psychotic dysfunctions.

Peciccia observes that functions elicited by affective touch (see 1A in Figure 1) are frequently impaired in individuals with psychosis (see 1B in Figure 1). However, as reviewed by Papi et al., the therapeutic potential of affiliative touch in psychosis remains largely unexplored, with only one case study reporting increased interoceptive accuracy, enhanced self-boundaries, and the elimination of delusional symptoms in a psychotic patient treated with an intervention called amniotic therapy. This intervention combines affiliative touch with early parent-child movement patterns in a warm water environment (Peciccia et al., 2022). Given these promising findings, further empirical research with larger samples of psychotic patients is essential.

Yang et al. conducted a randomized clinical trial to evaluate “lite touch,” a non-pharmacological intervention similar to affiliative touch, aimed at reducing anxiety and physiological stress in full-term women in labor. Participants were divided into an intervention group, receiving lite touch along with standard prenatal care, and a control group receiving standard care only. Anxiety was measured using the State Anxiety Inventory, while levels of cortisol and catecholamines in the saliva were used as markers of stress. Results showed that the intervention group had significantly reduced anxiety and stress markers post-intervention (*P* < 0.01), indicating lite touch's effectiveness in alleviating labor-related anxiety and promoting physiological stability.

Ugurlu and Keltner's examines how interpersonal touch serves as a form of emotion regulation. They highlight its essential role in emotional wellbeing, noting its efficacy in reducing negative emotions, distress, and pain across developmental stages and across species. Despite its benefits, the regulatory function of touch remains underexplored. The review discusses how touch modulates neurophysiological responses and communicates emotional states, with nuanced meanings across various relationships, from maternal bonds to brief interactions with strangers. They identify four key pathways by which touch supports emotional regulation, aiming to deepen understanding of its role in fostering emotional health.

Stupperich et al. conducted an investigation into spontaneous touch frequency in very preterm (VP) infants, an essential element of early motor development. Their comparative study revealed that VP infants exhibited a markedly lower frequency of spontaneous touches per minute relative to term infants. These findings indicate potential developmental discrepancies that may adversely impact motor skill acquisition in VP infants. The study underscores the necessity for longitudinal research to further elucidate motor developmental trajectories in this population, thereby informing the design of preventive and supportive interventions tailored for VP infants.
